# Self-administered EMDR therapy: potential solution for expanding the availability of psychotherapy for PTSD or unregulated recipe for disaster?

**DOI:** 10.1192/bjo.2020.92

**Published:** 2020-10-02

**Authors:** Lauren Z. Waterman, Maxwell Cooper

**Affiliations:** Mental Health Research UK MD(Res) student at King's College London, UK, RCPsych Working Group on the Health of Refugees and Asylum Seekers, and ST5 Higher Trainee in Psychiatry at the Maudsley Hospital, London, UK; academic general practitioner who has worked with asylum seekers and refugees in Glasgow and Brighton, UK

**Keywords:** Trauma, post-traumatic stress disorder, EMDR, self-help, refugees

## Abstract

**Background:**

Post-traumatic stress disorder (PTSD) carries a high disease burden worldwide, yet significant barriers exist to providing and accessing treatment for PTSD, particularly in refugee populations and in low- and middle-income countries. There is emerging evidence that self-administered psychological therapies, such as those accessed via online and mobile applications, are efficacious for many mental illnesses and increase access to treatment. Online and mobile applications offering self-help tools for eye movement desensitisation reprocessing (EMDR) therapy, an internationally recommended treatment for PTSD, are already widely distributed to the public.

**Aims:**

To present a commentary evaluating the potential benefits and risks of self-administered EMDR therapy: first, by conducting a search for existing peer-reviewed evidence relating to self-administered EMDR therapy; second, by presenting existing evidence for other self-help psychotherapies and evaluating what additional insight this could provide into the potential efficacy, safety, tolerability and accessibility of self-administered EMDR therapy; and, third, by describing the conflicting views of EMDR experts on the topic.

**Method:**

A search was conducted for articles related to internet, mobile, book or computerised self-help EMDR therapy. The following databases were searched systematically: Medline, PsycInfo, EMBASE, AMED, CINAHL, Psychology and Behavioural Sciences, Cochrane Database and the EMDR Library.

**Results:**

Only one small primary research study was found relating to self-administered EMDR therapy. The results indicated significantly reduced symptoms of PTSD, depression, anxiety, distress and disability between pre-treatment and 3 month follow-up. No serious adverse events were reported. However, substantial methodological issues were discovered.

**Conclusions:**

There is evidence that self-administered psychotherapies, in general, can be safe, effective and highly accessible. However, controversies persist regarding the safety and potential efficacy of self-administered EMDR therapy, and more robust research is needed. It is vital that methods are found to improve worldwide access to effective PTSD treatment, particularly given the current scale of migration to flee civil unrest.

## Eye movement desensitisation and reprocessing for post-traumatic stress disorder

Eye movement desensitisation and reprocessing (EMDR) therapy is recommended by many national and international bodies for the treatment of post-traumatic stress disorder (PTSD), including the UK's National Institute for Health and Clinical Excellence,^1^ the World Health Organization (WHO)^2^ and the American Psychiatric Association.^3^ The therapy involves the patient focusing on a traumatic image, thought or memory, as well as any associated body sensations or emotions, while concurrently receiving bilateral stimulation, which may be in the form of left-to-right eye movements or bilateral auditory stimulation.^4^ EMDR was developed by Francine Shapiro in 1989. There is no agreed mechanism by which this therapy improves the symptoms of PTSD;^5^ however, research has suggested that EMDR is more effective than pharmacological treatment and most other psychotherapies for treating PTSD. For example, a Cochrane review of psychological therapies for PTSD concluded that there was some evidence that EMDR and trauma-focused cognitive–behavioural therapy (CBT) were superior to non-trauma-focused CBT between 1 and 4 months following treatment, although the evidence was assessed as being low quality.^5^ Furthermore, two more recent meta-analyses comparing EMDR therapy with other therapies for PTSD found that EMDR was superior in reducing total PTSD scores,^6,7^ particularly for reducing intrusive thoughts and arousal severity,^6^ although studies were found to be at a high risk of bias.^7^

## The global burden of PTSD and access to treatment

### High-income countries

PTSD carries a high disease burden in the UK^1^ and across much of the world.^8^ A particularly high prevalence of PTSD within Western countries can be found in refugee and asylum seeker populations, in which many, prior to arrival in their destination country, have experienced interpersonal trauma in the form of imprisonment, torture or sexual assault.^8^ As an indicator of this burden: in 2017, 650 000 first-time asylum applications were made in the European Union (EU) member states alone,^9^ with a recent peak in 2015 of 1.2 million first-time applications to EU countries; and cross-sectional studies have indicated that the prevalence of PTSD in Western countries is more than 17% in asylum seekers^8^ and 9% in refugees.^10^

PTSD is also associated with a high economic burden in high-income countries,^11^ since it causes a considerable loss of human capital^12,13^ and the costs of treating PTSD with psychological therapy can be high.^1,14^ Furthermore, there are significant barriers to providing and accessing psychological therapy for PTSD, including: perceived stigma associated with psychological therapy;^15^ physical constraints such as living in a sparse population, agoraphobia, poor physical mobility or lack of access to transportation;^16^ rationing of services according to symptom severity or degree of functional impact; high therapy costs; and long waiting lists for psychotherapy within national health services. In some countries, waiting lists for psychotherapy may be growing longer: for example, in a survey of 2026 psychotherapists in the UK in 2014, 57% reported an increase in waiting times for psychotherapy from 2013 to 2014, 77% reported an increase in clients with complex needs and 29% reported a higher case-load during 2014 compared with the previous year.^17^ Moreover, refugees and asylum seekers can face additional significant barriers to accessing healthcare and psychological therapies, including a lack of awareness of entitlement, language barriers,^18^ repeated relocation within the country^19^ and a lack of trust in healthcare professionals.^20^

### Low- and middle-income countries

Trauma from interpersonal violence, particularly torture, carries the highest risk of a person developing PTSD.^21^ This association was supported by Steel and colleagues in a large review and meta-analysis of survey studies comprising a population of 81 866, in which the authors concluded that torture was the strongest substantive factor associated with PTSD.^22^ In many low- and middle-income countries (LMICs) in which widespread conflict is rife, torture is a risk factor that can be considered endemic.^23^ Unfortunately, most people with serious mental illness in low-resource settings do not have access to efficacious treatment,^24,25^ owing to a shortage of available resources, inefficiencies in the use of those resources and inequitable distribution of services, both between and within communities.^25^ Furthermore, for those who are able to access treatment for mental illness in LMICs, stigma and fear of discrimination associated with help-seeking can act as additional barriers.^25^ Accordingly, the WHO's World Mental Health Survey Consortium found that 76–85% of the people surveyed from 2001 to 2003 with serious mental illness in LMICs did not receive any treatment;^26^ similar results were reported by a study of the 2001–2012 WHO Mental Health Surveys.^27^ Consequently, many leaders in global mental health have called for urgent action towards the development and evaluation of mobile and internet technologies to overcome these barriers and thus increase access to psychological therapies worldwide.^23^

## The digital era of healthcare

The World Wide Web contains a maze of regulated and unregulated health websites, specialist and patient chat forums and YouTube videos; now, mobile phone and tablet applications expand the pool of options, inflating the complexity of assessing and regulating the quality of information available.^28^ Although in the USA, medical mobile applications may be partially regulated by the Food and Drug Administration,^29^ in many countries, including the UK, these remain unregulated. In a recent World Psychiatry article, mHealth experts urgently called for universal standards for mental health app quality control.^30^ Furthermore, whereas some websites are run and regulated by specialist healthcare agencies or carry a mark of conforming to quality standards, such as the ‘HON Code’ (Health on the Net Foundation),^31^ many more are unregulated. The public are increasingly relying on e-Health as a source of medical information,^32^ but lay people may not know how to adequately assess whether a website or app is trustworthy.^33^

## Are the public already self-administering EMDR therapy?

This calls into question whether the public are already self-administering EMDR therapy. Online health forums can provide a useful indication of how members of the public are already using tools for self-help therapy. Online forums such as ‘my PTSD’ (www.myptsd.com) show PTSD sufferers discussing the prospect of self-administered EMDR, with many users declaring they had already tried this at home. For example, in a thread entitled ‘A Recipe for Self-EMDR’,^34^ one user commented that although she was unsure whether self-administered EMDR was a good idea, she considered it ‘better than the alternative of paralyzing re-experiencing’ when she did not have access to an EMDR therapist; another user commented that he or she had used every resource possible to escape from their ‘PTSD prison’. Users appeared to be not only using visual EMDR tools but also attempting to create bilateral auditory stimulation using audio files, and using the ‘butterfly hug’ for bilateral tactile stimulation. Conversely, other users set out to warn of potential risks, explaining that their therapists have advised them against engaging in trauma processing without a practitioner.

A search of these stores unearthed 11 Apple and eight Android apps already in existence that claim to offer tools for self-administered EMDR therapy. Apple does not currently offer information regarding the number of application downloads; however, for the Android apps, the number of downloads per app ranged from ‘50+’ to ‘7000+’. In addition, a brief search of the internet for ‘self-help EMDR’ reveals a plethora of unregulated websites advertising tools for self-help EMDR therapy for public use.

## Aims and objectives of this study

Based on exploration of online forums, self-help book reviews and the download counts of self-help EMDR mobile applications, it appears that members of the public are already attempting self-administered EMDR therapy using the available resources. Therefore, the aim of this article was to present a commentary evaluating the potential risks and benefits of self-administered EMDR therapy by:

- conducting a search for existing peer-reviewed evidence relating to self-administered EMDR therapy;
- presenting existing evidence for other self-help psychotherapies, in both low- and high-income countries, and evaluating what additional insight this could provide into the potential efficacy, safety, tolerability and accessibility of self-administered EMDR therapy; and
- describing the conflicting views of EMDR experts on the topic.

## Methods

A systematic search was conducted on published peer-reviewed articles related to internet, mobile, book or computerised self-help EMDR therapy. PRISMA guidelines were adhered to, and there were no exclusion criteria. The search terms were in English; however, articles retrieved in other languages were included. The following databases were searched systematically: Medline, PsycInfo, EMBASE, AMED, CINAHL, Psychology and Behavioural Sciences, and Cochrane Controlled Register of Trials. The search included all peer-reviewed articles up to November 2018.

**Fig. 1:**
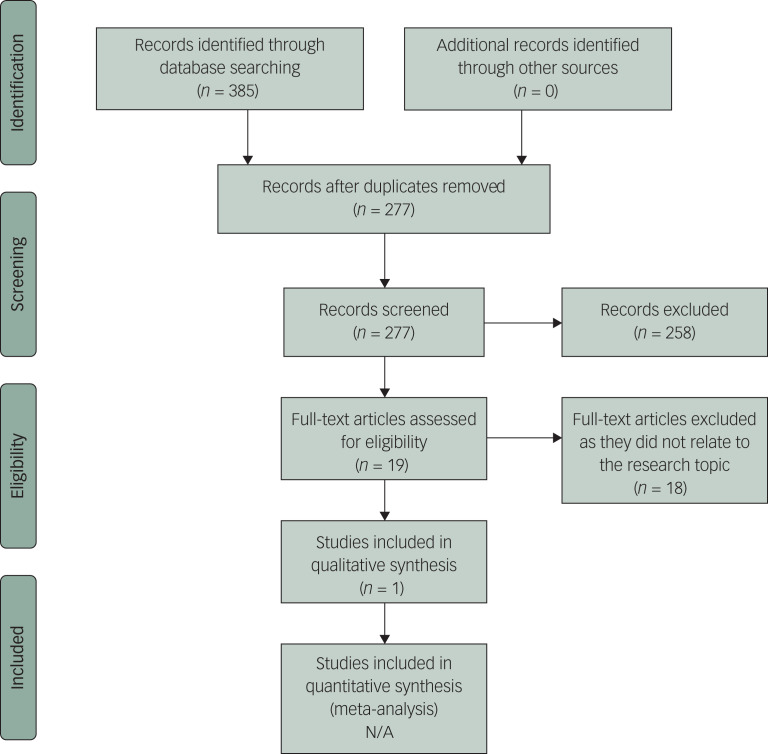
PRISMA 2009 flow diagram

The search of the academic literature was conducted in the title/abstract field with the following keywords and MeSH terms: (EMDR OR eye movement desensitization OR eye movement desensitisation OR exp Eye Movement Desensitization Reprocessing) AND (comput* OR online OR internet OR iEMDR OR cEMDR OR c-EMDR OR telemedicine OR exp Telemedicine OR telehealth OR telepsychiatry OR software OR smartphone OR ‘cell phone’ OR ‘cellular phone’ OR eHealth OR self* OR e-* OR app OR phone OR mobile OR mHealth OR m-health OR ‘mobile health’ OR mLearning OR telehealth OR website OR world wide web). The full search strategy can be found in Table 1, and a PRISMA flow diagram is shown in Fig. 1.

**Table 1:** Full search strategy

Healthcare Databases Advanced Search (HDAS) Export			
#	Database	Search term	Results
2	Medline	“EYE MOVEMENT DESENSITIZATION REPROCESSING”/	145
3	Medline	(EMDR OR “eye movement desensitization” OR “eye movement desensitisation”).ti,ab	534
4	Medline	(2 OR 3)	555
5	Medline	(comput* OR online OR telemedicine OR eHealth OR self* OR app OR apps OR phone OR mobile OR mHealth OR m-health OR “mobile health” OR mLearning OR telehealth OR website* OR “world wide web” OR Internet).ti,ab	1 345 308
6	Medline	TELEMEDICINE/	16 655
7	Medline	SOFTWARE/	95 376
8	Medline	“MOBILE APPLICATIONS”/ OR SMARTPHONE/ OR “CELL PHONE”/	10 129
9	Medline	INTERNET/	63 231
10	Medline	“SELF CARE”/	29 770
11	Medline	“SELF ADMINISTRATION”/	10 410
12	Medline	(5 OR 6 OR 7 OR 8 OR 9 OR 10 OR 11)	1 454 361
13	Medline	(4 AND 12)	94
14	CINAHL	(EMDR OR “eye movement desensitization” OR “eye movement desensitisation”).ti,ab	156
15	CINAHL	“EYE MOVEMENT DESENSITIZATION AND REPROGRAMMING”/	129
16	CINAHL	(14 OR 15)	232
17	CINAHL	(comput* OR online OR telemedicine OR eHealth OR self* OR app OR apps OR phone OR mobile OR mHealth OR m-health OR “mobile health” OR mLearning OR telehealth OR website* OR “world wide web” OR Internet).ti,ab	264 899
18	CINAHL	“WORLD WIDE WEB”/	56 677
19	CINAHL	INTERNET/	28 688
20	CINAHL	TELEPSYCHIATRY/	153
21	CINAHL	TELEHEALTH/	3949
22	CINAHL	TELEMEDICINE/	4236
23	CINAHL	SOFTWARE/	16 011
24	CINAHL	“MOBILE APPLICATIONS”/	1960
25	CINAHL	SMARTPHONE/	830
26	CINAHL	“CELLULAR PHONE”/	558
27	CINAHL	“SELF CARE”/	24 379
28	CINAHL	“SELF ADMINISTRATION”/	2084
29	CINAHL	(17 OR 18 OR 19 OR 20 OR 21 OR 22 OR 23 OR 24 OR 25 OR 26 OR 27 OR 28)	353 981
30	CINAHL	(16 AND 29)	30
31	AMED	(EMDR OR “eye movement desensitization” OR “eye movement desensitisation”).ti,ab	37
32	AMED	(comput* OR online OR telemedicine OR eHealth OR self* OR app OR apps OR phone OR mobile OR mHealth OR m-health OR “mobile health” OR mLearning OR telehealth OR website* OR “world wide web” OR Internet).ti,ab	22 266
33	AMED	“SELF CARE”/	1701
34	AMED	TELEMEDICINE/	636
35	AMED	SOFTWARE/	354
36	AMED	INTERNET/	998
37	AMED	COMPUTERS/	1462
38	AMED	“COMPUTER SYSTEMS”/	274
39	AMED	(32 OR 33 OR 34 OR 35 OR 36 OR 37 OR 38)	24 225
40	AMED	(31 AND 39)	6
41	EMBASE	(EMDR OR “eye movement desensitization” OR “eye movement desensitisation”).ti,ab	765
42	EMBASE	“EYE MOVEMENT DESENSITIZATION REPROCESSING”/	119
43	EMBASE	“EYE MOVEMENT DESENSITIZATION AND REPROCESSING”/	118
44	EMBASE	(41 OR 42 OR 43)	807
45	EMBASE	(comput* OR online OR telemedicine OR eHealth OR self* OR app OR apps OR phone OR mobile OR mHealth OR m-health OR “mobile health” OR mLearning OR telehealth OR website* OR “world wide web” OR Internet).ti,ab	1 822 171
46	EMBASE	“SELF HELP”/	12 761
47	EMBASE	“SELF CARE”/	46 076
48	EMBASE	exp TELEMEDICINE/	29 856
49	EMBASE	TELEHEALTH/	3515
50	EMBASE	SMARTPHONE/	4240
51	EMBASE	“MOBILE PHONE”/	13 602
52	EMBASE	SOFTWARE/	25 162
53	EMBASE	“MOBILE APPLICATION”/	5222
54	EMBASE	INTERNET/	94 997
55	EMBASE	(45 OR 46 OR 47 OR 48 OR 49 OR 50 OR 51 OR 52 OR 53 OR 54)	1 916 787
56	EMBASE	(44 AND 55)	150
57	PsycINFO	(EMDR OR “eye movement desensitization” OR “eye movement desensitisation”).ti,ab	1685
58	PsycINFO	“EYE MOVEMENT DESENSITIZATION THERAPY”/	1296
59	PsycINFO	(57 OR 58)	1734
60	PsycINFO	(comput* OR online OR telemedicine OR eHealth OR self* OR app OR apps OR phone OR mobile OR mHealth OR m-health OR “mobile health” OR mLearning OR telehealth OR website* OR “world wide web” OR Internet).ti,ab	673 559
61	PsycINFO	“SELF-HELP TECHNIQUES”/	3929
62	PsycINFO	“SELF-MANAGEMENT”/	5910
63	PsycINFO	INTERNET/	33 168
64	PsycINFO	“COMPUTER ASSISTED THERAPY”/	921
65	PsycINFO	TELEMEDICINE/	4933
66	PsycINFO	“CELLULAR PHONES”/	3873
67	PsycINFO	INTERNET/	33 168
68	PsycINFO	(60 OR 61 OR 62 OR 63 OR 64 OR 65 OR 66 OR 67)	681 322
69	PsycINFO	(59 AND 68)	291
70	PsycINFO	69 [Peer reviewed]	172
71	PsycINFO	69 [Record type Dissertation Abstract]	41

Psychology and Behavioral Sciences Collection Export				
#	Query	Limiters/Expanders	Last Run Via	Results
S1	TI ( EMDR OR “eye movement desensitization” OR “eye movement desensitisation”) ) OR AB ( EMDR OR “eye movement desensitization” OR “eye movement desensitisation”) )	Search modes - Boolean/Phrase	Interface - EBSCOhost Research Databases Search Screen - Advanced Search Database - Psychology and Behavioral Sciences Collection	181
S2	SU EYE MOVEMENT DESENSITIZATION REPROCESSING	Search modes - Boolean/Phrase	Interface - EBSCOhost Research Databases Search Screen - Advanced Search Database - Psychology and Behavioral Sciences Collection	142
S3	S1 OR S2	Search modes - Boolean/Phrase	Interface - EBSCOhost Research Databases Search Screen - Advanced Search Database - Psychology and Behavioral Sciences Collection	202
S4	TI ( comput* OR online OR telemedicine OR eHealth OR self* OR app OR apps OR phone OR mobile OR mHealth OR m-health OR “mobile health” OR mLearning OR telehealth OR website* OR “world wide web” OR Internet ) OR AB ( comput* OR online OR telemedicine OR eHealth OR self* OR app OR apps OR phone OR mobile OR mHealth OR m-health OR “mobile health” OR mLearning OR telehealth OR website* OR “world wide web” OR Internet )	Search modes - Boolean/Phrase	Interface - EBSCOhost Research Databases Search Screen - Advanced Search Database - Psychology and Behavioral Sciences Collection	117,265
S5	DE “INTERNET”	Search modes - Boolean/Phrase	Interface - EBSCOhost Research Databases Search Screen - Advanced Search Database - Psychology and Behavioral Sciences Collection	2,745
S6	(DE “TELEMEDICINE”) OR (DE “TELECOMMUNICATION in psychiatry” OR DE “TELECOMMUNICATION in psychotherapy”)	Search modes - Boolean/Phrase	Interface - EBSCOhost Research Databases Search Screen - Advanced Search Database - Psychology and Behavioral Sciences Collection	401
S7	DE “MOBILE apps” OR DE “ENTERTAINMENT mobile apps” OR DE “IPHONE mobile apps” OR DE “MOBILE apps in business” OR DE “MOBILE apps in education” OR DE “NAVIGATION & travel mobile apps” OR DE “ONLINE dating mobile apps” OR DE “PHYSICAL fitness mobile apps” OR DE “READING mobile apps” OR DE “SHOPPING mobile apps” OR DE “SOCIAL networking mobile apps” OR DE “TABLET mobile apps” OR DE “WEATHER forecasting mobile apps” OR DE “MOBILE computing software” OR DE “POCKET computer software”	Search modes - Boolean/Phrase	Interface - EBSCOhost Research Databases Search Screen - Advanced Search Database - Psychology and Behavioral Sciences Collection	342
S8	S4 OR S5 OR S6 OR S7	Search modes - Boolean/Phrase	Interface - EBSCOhost Research Databases Search Screen - Advanced Search Database - Psychology and Behavioral Sciences Collection	117,828
S9	S3 AND S8	Search modes - Boolean/Phrase	Interface - EBSCOhost Research Databases Search Screen - Advanced Search Database - Psychology and Behavioral Sciences Collection	22

Cochrane Controlled Register of Trials Export		
ID	Search	Hits
#1	(comput* OR online OR telemedicine OR eHealth OR self* OR app OR apps OR phone OR mobile OR mHealth OR m-health OR “mobile health” OR mLearning OR telehealth OR website* OR “world wide web” OR Internet):ti,ab,kw (Word variations have been searched)	142 227
#2	MeSH descriptor: [Telemedicine] explode all trees	1948
#3	MeSH descriptor: [Self Care] explode all trees	5096
#4	MeSH descriptor: [Self Administration] explode all trees	714
#5	#1 or #2 or #3 or #4	142 326
#6	(EMDR OR “eye movement desensitization” OR “eye movement desensitisation”):ti,ab,kw (Word variations have been searched)	234
#7	MeSH descriptor: [Eye Movement Desensitization Reprocessing] explode all trees	46
#8	#6 or #7	234
#9	#5 AND #8	42

In addition, Francine Shapiro's EMDR Library, run by the EMDR International Association (EMDRIA), was searched using the same search strategy, using both the ‘journal’ and ‘publication’ search options. The Study Register (via http://clinicaltrials.gov) was also searched. Furthermore, a snowball search was conducted for the identification of further potentially relevant studies by screening the reference lists of articles relating to the topic, and Web of Science was used to check for any other primary research articles citing any relevant articles that were found.

## Results

A total of 277 articles were retrieved, following the removal of duplicates, from the search of seven databases (Medline, PsycInfo, EMBASE, AMED, CINAHL, Psychology and Behavioural Sciences, and Cochrane Controlled Register of Trials). Of these, only one relevant article was found relating to research into the efficacy, tolerability, feasibility or safety of self-help EMDR therapy;^35^ no additional relevant articles were retrieved from Francine Shapiro's EMDR library, the Study Register, the snowball search, or the search of Web of Science for any other articles citing this one. The relevant article, published in 2013, described a small quasi-experimental feasibility study based in Australia, which aimed to explore the acceptability and efficacy of a six-lesson internet-delivered treatment that combined elements of EMDR therapy (iEMDR) and internet CBT (iCBT), alongside weekly telephone and email contact with a clinical psychologist. The study used an open trial design with 15 participants who met clinical criteria for PTSD and no control group.

The results indicated significantly reduced symptoms between pre-treatment, post-treatment and 3 month follow-up, according to an analysis of completers using paired-sample *t*-tests. Large effect sizes were found for PTSD according to the PTSD Symptoms Scale-Interview (PSS-I) questionnaire (t10 = 3.66, *P* = 0.004) and PTSD Checklist-Civilian Version (PCL-C) questionnaire (t10 = 2.73, *P* = 0.021) between pre- and post-treatment, and between pre-treatment and follow-up (PSS-I: t10 = 4.90, *P* = 0.001; PCL-C: t10 = 4.26, *P* = 0.002). Large effect sizes were also found for measures of distress and anxiety; and moderate effect sizes for depression and disability. By the end of treatment, 55% of participants no longer met the criteria for PTSD; this was sustained at follow-up. Effect sizes were slightly lower when intention-to-treat analyses were used. However, the absence of a control group meant that any improvement in symptoms may have been due to other factors such as the passing of time since the traumatic event or repeated measurement effects.

The above study also sought to investigate the acceptability and risks of iEMDR therapy using self-report measures of symptom severity and adverse events. Of the 11 participants who completed post-treatment assessments, nine reported that they would recommend the course to a friend with PTSD. However, eight participants reported increases in re-experiencing symptoms following iEMDR, and three participants reported an overall worsening of symptoms at the end of treatment, although these had improved by follow-up. The authors argued that this symptom exacerbation was no higher than that which had previously been reported for waiting-list participants with PTSD, and also no higher than the error rates of the instruments used to detect adverse events. No *serious* adverse events were reported by any participants, defined as hospital admissions, suicide attempts or onset of substance misuse. However, the attrition rate was high at 4/15; furthermore, four participants (a mixture of those who had dropped out and those who had completed the course of treatment) did not complete any post-treatment assessments; therefore, it was not possible to determine whether or not these participants experienced any serious adverse effects. Additional limitations of the study included its small sample size and lack of control group, and a study design that did not allow evaluation of whether effects were due to the iCBT or iEMDR components of the treatment. Also, as those who were not ‘residents’ of Australia were excluded, the effectiveness and safety of the treatment for non-residents, such as asylum seekers with insecure immigration status, could not be determined.

## Discussion

### The search for research into self-administered EMDR therapy

Although there are already many self-help tools for EMDR therapy available via books and technology, with evidence that members of the public are already using these tools, the only published trial of any self-help EMDR therapy is an open trial of an internet-delivered guided self-help programme. That trial suggested that the iEMDR programme may be safe and efficacious; however, significant methodological flaws preclude firm conclusions from being drawn, including the small sample size, high attrition rate and absence of a control group. Furthermore, participants in that trial had weekly contact with a clinical psychologist so were not left to do their therapy completely unsupervised: therefore, conclusions cannot be made about the safety of unsupervised self-help EMDR therapy. A larger study evaluating the efficacy and potential adverse effects of the iEMDR therapy programme is required, using a randomised controlled trial (RCT) design. Changes to the programme may also be required, as the protocol used in the open trial was only moderately tolerated by the participants who completed post-treatment assessments. For example, past studies have indicated that including more social interactive elements such as an accompanying virtual human avatar throughout treatment may be beneficial;^36^ this might improve tolerability of the intervention.

Although there are numerous mobile phone applications offering tools for self-administered EMDR therapy, no article was found on EMDR therapy delivered via mobile phone applications during the systematic literature search. Therefore, the efficacy and risks of EMDR self-administration using mobile phones is currently unknown. This lack of peer-reviewed literature is a key gap, particularly given the unknown effectiveness of EMDR when it is conducted using a screen as small as a mobile phone's (where the left and right dot positions may be less than an inch apart on screen, which we speculate could affect treatment outcomes).

### The evidence for other self-help psychotherapies

The growing issue of poorly regulated and unregulated health websites and applications is highlighted above. On the other hand, however, there may be benefits to the use of internet, computerised and mobile applications for delivering psychological therapies in the form of self-help or guided self-help programmes. There is already some evidence in the peer-reviewed literature that internet-delivered psychological therapies can bypass some of the barriers to accessing treatment mentioned above, while being safe and cost-effective for a range of different mood, anxiety and substance-misuse disorders.^37^ For example, internet-guided self-help therapies for anxiety disorders that include exposures to anxiety-inducing stimuli, such as FearFighter for the treatment of phobias and panic, have been shown to be safe and effective.^38^ In addition, a cluster RCT of a self-help programme for trauma-exposed refugees in Uganda, guided only by briefly trained lay facilitators, demonstrated good feasibility and safety profiles for the intervention and indicated its effectiveness for reducing psychological distress in a population otherwise unlikely to receive formal psychological support.^39^

A meta-analysis of internet-based psychological therapies for PTSD (mostly using cognitive–behavioural models) demonstrated an emerging evidence base supporting e-Mental Health to treat symptoms of PTSD.^16^ There have also been initially promising results of research into the quality and effectiveness of medical mobile applications in the treatment of mental health disorders, such as ‘PTSD Coach’, a mobile application comprising self-help techniques including relaxation for PTSD treatment that has already been downloaded by over 100 000 users across 74 countries worldwide.^40^ A recent meta-analysis of six studies into these applications for PTSD treatment (of which five used PTSD Coach) found modest pre-post effect sizes for reduction in PTSD and depressive symptoms, although analysis of the two RCTs suggested the intervention was not superior to a waiting-list control.^41^

Although many self-help treatments have been shown to be effective, sustaining patient engagement may be problematic, and therapy completion rates as low as 20% have been reported for online CBT.^42^ However, as the costs of self-help therapies are comparatively low, a large number of people may be treated at low cost, so the potential gain in care can still be substantial despite high drop-out rates. The limited existing literature suggests that online therapy does not discourage or affect the efficacy of future personalised therapy,^43^ although further research into this is needed.

Access to technology worldwide is increasing rapidly, including in LMICs. In a 2015 survey of 21 emerging and developing countries, a median of 54% reported using the internet and 37% reported owning a smartphone (Pew Research Centre, 2016),^44^ and there is a growing body of evidence that treatments using mobile and internet technologies can be feasible and efficacious in LMICs, while increasing the availability and accessibility of mental health services.^23,45^

### Potential risks and controversies around self-administered EMDR therapy

A session of EMDR therapy can lead to a short-term increase in distress for patients,^46,47^ which Francine Shapiro, the founder of EMDR therapy, warned could lead to a transient increase in suicidality in some patients.^47^ She explained that this may be due to the resurfacing of dissociated information, emotions and physical sensations. Therefore, according to the treatment protocol, therapists first assess their patients’ suitability and readiness for EMDR therapy prior to commencing the active processing of memories, particularly by evaluating their abilities to successfully utilise self-control and relaxation techniques and to maintain their own safety.^47^ Shapiro further cautioned that EMDR processing may even be ‘very disturbing’^47^ (p. 89) and that ‘the lack of adequate screening, preparation, or implementation of EMDR can have literally fatal consequences’^47^ (p. 303). The EMDR UK and Ireland Association also advised that ‘according to American Psychological Association ethical guidelines, all prescribed therapies should be done according to the standardised procedures that have been examined by research’.^46^

However, it appears that these warnings may have been drawn solely from anecdotal evidence; it has been argued that previous research studies have not assessed or reported upon the adverse effects of EMDR therapy with enough rigor to be able to make a conclusion about the risk of harm.^48^ In particular, there is inadequate evidence in the published literature to either prove or disprove that computerised, mobile or self-help EMDR therapy can be harmful. Shapiro co-authored one article in 2000 in which EMDR therapists were described as ‘escorts rather than active agents during therapy’,^49^ (p. 190) a notion that that brings into question whether digital ‘escorts’ could be substituted for individual therapists.

Opinions differ among the authors of the self-help literature on the potential safety of self-administered EMDR therapy. EMDR therapists – and, indeed, Shapiro herself – have begun to publish self-help books using techniques derived from EMDR therapy with the aim of reducing readers’ symptoms of PTSD, anxiety or insomnia, among other conditions.^50,51^ For example, Shapiro's self-help book recommends the unsupervised use of several aspects of the preparation phase of EMDR therapy, including affect scan, identifying unprocessed memories and negative cognitions, and breathing techniques.^51^ However, Shapiro cautions that the memory processing itself should not be done without following all the EMDR therapy procedures with a trained therapist, and that professional help should be sought if symptoms are severe or persistent. On the other hand, self-help books are also available to the public that recommend that all eight stages of EMDR therapy in their entirety can be safely tried by members of the public unsupervised.^52,53^ Concerningly, the author of one of these books states that they are not themselves trained in EMDR therapy,^52^ and the authors of the other book declare, without citing scientific evidence, that self-help EMDR is as safe as ‘grandma gently moving backwards and forwards in her rocking chair’^53^ (p. 1).

Further to the paucity of research and the lack of consensus among EMDR therapists about the efficacy and safety of self-administered EMDR, it is concerning that poor-quality and unregulated mobile apps and internet programmes, as well as self-help books, are so easily available to the public. The mean quality of nine applications advertising themselves as tools for self-help EMDR therapy for PTSD was recently rated as 2.91 using the Mobile Application Rating Scale German Version (MARS-G), indicating ‘poor’ quality.^54^ To our knowledge, the quality of internet websites providing these tools has yet to be rated.

### Strengths and limitations

This was the first commentary or review article that has explored the risks and benefits of self-help, computerised, internet or mobile EMDR therapy, and how these could potentially improve accessibility to EMDR therapy for treatment of PTSD in low- and high- income countries. A strength of this study is the thorough systematic search that was conducted for peer-reviewed articles on the topic, using several search engines. However, the main limitation was the lack of available primary research published on the topic, which limited the analysis and the conclusions that could be drawn. In addition, other than the search of the EMDR Library, it did not include any further search of the grey literature.

### Conclusions

EMDR is recommended by many national and international bodies for the treatment of PTSD. There is already evidence from RCTs and reviews that online, mobile and other self-administered psychological therapies for a range of psychiatric disorders, including CBT for PTSD, can bypass some of the barriers to accessing psychological therapy, while being safe and effective. Many of these are already being used worldwide to increase access to psychological therapies, in both low- and high-income countries. In addition, it appears that applications from private companies and books advertising tools for self-help EMDR therapy have already been made widely available to the public. It is therefore surprising that so little research has been conducted into this self-help therapy thus far.

There are conflicting views presented by authors of the EMDR and self-help literature about the potential effectiveness and safety of self-administered EMDR therapy; however, despite warnings from Francine Shapiro herself about the potential risks, there has been no published research evidence to suggest that these warnings are well founded. Furthermore, the limited evidence available suggests that self-administered EMDR therapy for PTSD *may* be safe and efficacious. Therefore, further research is warranted to assess the effectiveness and risks associated with self-administered EMDR therapy using computerised, online or mobile application tools, and to determine whether these may be cost-effective means for improving the availability and accessibility of EMDR therapy, across cultures and in both low- and high-income countries.
