# Self-administered EMDR therapy: potential solution for expanding the availability of psychotherapy for PTSD or unregulated recipe for disaster? – ERRATUM

**DOI:** 10.1192/bjo.2020.128

**Published:** 2020-10-29

**Authors:** Lauren Z. Waterman, Maxwell Cooper

Unfortunately, a mistake was made by the publisher, which resulted in several amendments requested by the authors at the proofs stage not being acted upon prior to publication. These amendments are as follows:

- Line 57 “in 2017, 650 000 first-time asylum applications…” should read “in 2019, 612 700 first-time asylum applications…” and the associated reference 9 should have been changed to: Eurostat. *Asylum statistics*. Eurostat statistics explained, 2020 (https://ec.europa.eu/eurostat/statistics-explained/index.php/Asylum_statistics#Age_and_gender_of_first-time_applicants [cited 13 Sept 2020])
- Line 87 “A search of these stores unearthed 11 Apple and eight Android…” should read “Additionally, a search of mobile application stores unearthed 11 Apple and eight Android…”
- Line 93 “In many low- and middle income countries (LMICs) in which widespread conflict is rife, torture is a risk factor that can be considered endemic” should read “In many low- and middle-income countries (LMICs) experiencing war or civil unrest, torture is a risk factor that can be considered endemic”
- Line 182 “less than an inch” should read “less than a few inches”
- Line 418 “and the authors of the other book declare…” should read “and the authors of another book declare…”

The publisher apologises for these errors.
